# The relationship between MnSOD Val16Ala gene polymorphism and the level of serum total antioxidant capacity with the risk of chronic kidney disease in type 2 diabetic patients: a nested case-control study in the Tehran lipid glucose study

**DOI:** 10.1186/s12986-018-0264-0

**Published:** 2018-04-11

**Authors:** Mehrnaz Abbasi, Maryam S. Daneshpour, Mehdi Hedayati, Azadeh Mottaghi, Katayoun Pourvali, Fereidoun Azizi

**Affiliations:** 1 Department of Cellular and Molecular Nutrition, National Nutrition and Food Technology Research Institute, Faculty of Nutrition Science and Food Technology, Shahid Beheshti University of Medical Sciences, Tehran, Iran; 2 Department of Nutritional Sciences, Texas Tech University, Lubbock, TX USA; 3 Cellular Molecular and Endocrine Research Center, Research Institute for Endocrine Sciences, Shahid Beheshti University of Medical Sciences, Tehran, Iran; 4 Research Center for Prevention of Cardiovascular diseases, Institute of endocrinology & metabolism, Iran University of Medical Sciences, Tehran, Iran; 5 Nutrition and Endocrine Research Center, Research Institute for Endocrine Sciences, Shahid Beheshti University of Medical Sciences, Tehran, Iran; 6 Endocrine Research Center, Research Institute for Endocrine Sciences, Shahid Beheshti University of Medical Sciences, Tehran, Iran

**Keywords:** Manganese mitochondrial superoxide dismutase (MnSOD), Val16Ala polymorphism, Type 2 diabetes mellitus, Chronic kidney disease, Total antioxidant capacity (TAC)

## Abstract

**Background:**

Several studies have shown significant associations between manganese superoxide dismutase (MnSOD) Val16Ala polymorphism and diabetic complications, but this association has not been explored in relation with chronic kidney disease (CKD) in Type 2 diabetes mellitus (T2DM) patients. Total antioxidant capacity (TAC) level changes in diabetic condition and may play important role in onset or progression of the disease and its complications. The present study investigated the association of MnSOD Val16Ala polymorphism and serum TAC with the risk of CKD in T2DM patients.

**Methods:**

This nested case-control study included 280 type 2 diabetic patients with CKD and 280 age, sex and diabetes duration-matched control subjects selected from the participants of the Tehran Lipid and Glucose Study. MnSOD val16Ala (rs4880) SNP was genotyped by the Tetra-Primer ARMS-polymerase chain reaction analysis. Serum TAC was measured using ferric-reducing antioxidant power assay. Statistical analysis was performed using STATA statistical package v.12.0 or SPSS (Version 22.0).

**Results:**

The Ala allele of the MnSOD Val16Ala polymorphism was associated with a lower risk of CKD (odds ratio (OR), 0.55; 95% confidence interval (CI), 0.36–0.84; *P* = 0.006). Median serum TAC in CKD group was 920 μmol/L and was significantly lower (*p* < 0.001) compared to the control group (1045 μmol/L). Using an adjusted conditional logistic regression, we didn’t observe any significant interaction between MnSOD Val16Ala SNP with quartiles of serum TAC in relation to CKD.

**Conclusion:**

A significant association was found between the MnSOD Val16Ala polymorphism and CKD, but this association is not affected by serum TAC level in T2DM patients.

## Background

**Fig. 1:**
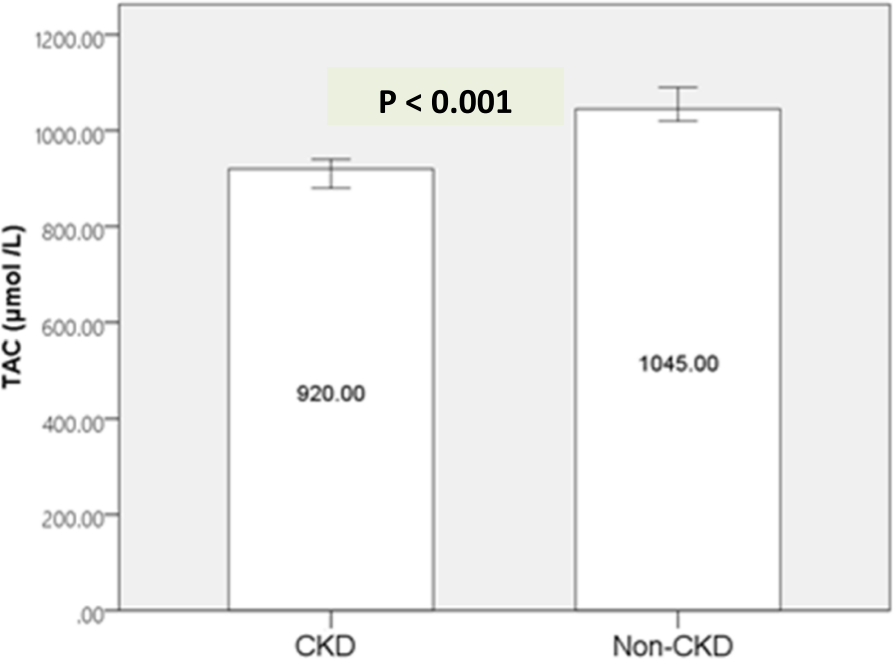
Median serum total antioxidant capacity (TAC) in CKD group and non-CKD group. Bars show Median and error bars show 95%CI. Mann–Whitney U test *P* value

Type 2 diabetes mellitus (T2DM) is the leading cause of chronic kidney disease (CKD) and an important health problem with increasing prevalence worldwide [1–3]. Approximately 50% of patients with T2DM suffer from a moderate-to-severe form of CKD (glomerular filtration rate (GFR) < 60 ml/min/1.73m^2^) [2, 3]. CKD and diabetes are prominent risk factors of cardiovascular disease and all three qualify as multiple morbidities [4]. In addition, the high treatment cost of diabetes markedly increases in the presence of chronic complications such as CKD [5, 6] (Fig. 1).

Oxidative stress is the excess formation and/or insufficient removal of highly reactive molecules such as reactive oxygen species (ROS) and is induced by elevation in glucose and free fatty acid (FFA) levels. It plays an important role in the pathogenesis and progression of T2DM as well as CKD [7, 8]. Mitochondrial respiratory system impairment increases oxidative stress in T2DM patients. Normal cell function is inhibited by increased production of ROS and impairment of the antioxidant defense mechanism by damage to cell biomolecules. CKD can become advanced due to a significant increase in the generation of ROS [4].

Manganese superoxide dismutase (MnSOD) is a key enzyme in antioxidant defense systems and the most important member of the SOD family which plays a crucial role fighting mitochondrial superoxide radicals [9]. There is growing evidence that genetic variants are essential factors in the pathogenesis and development of DM and its complications [10, 11]. Recently, genome-wide association studies have identified more than 260 single-nucleotide polymorphisms (SNPs) in relation to T2DM [12]. It is also generally accepted that there is a genetic susceptibility to CKD [11]. Previous studies have identified antioxidant gene variants and risk genotypes in diabetic populations of different ethnicities [7, 13, 14].

Chromosome 6q25 is the host for the MnSOD gene. Because they maintain cellular ROS levels, structural and/or functional SNPs of the MnSOD encoding gene are prominent fronts in the defense against ROS production [4]. A number of polymorphisms in this sequence have been described, but only the Val16Ala has demonstrated to have a functional significance [7, 13, 15]. The SNP rs4880 has been identified at the 16th amino acid position on the second exon [7, 13, 14, 16].

MnSOD activity is affected by functional Val16Ala polymorphism through structural changes in the β-sheet to α-helix in the mitochondrial targeting domain, which can cause a 30% to 40% increase in MnSOD activity in mitochondria. The presence of Valine (T allele) leads to the production of instable mRNA and reduces transport of the enzyme into the mitochondrial matrix and then its antioxidant function. This could influence the severity of oxidative stress related to diabetes and its chronic complications [9, 16–18]. Given the close link between CKD and T2DM, it is plausible that T2DM-related antioxidant gene variants and risk genotypes may be involved in the progression of CKD [6, 17].

The cumulative antioxidant capacity of all antioxidants is calculated as the serum TAC [19, 20]*.* Oxidative stress and the delay and prevention of its complications result from TAC modification. Although not consistent, the majority of research indicates possible modulation of the MnSOD Val16Ala SNP by different factors.

The probable interaction between Val16Ala SNP of the MnSOD gene and health-related factors such as serum TAC remains an open question [13]. The association between MnSOD Val16Ala (rs4880) and the risk of CKD in DM patients has not yet been clarified. To the best of our knowledge, this is one of the first studies to examine the relation of polymorphisms in Iranian T2DM patients. The present case-control study was designed to investigate the association of MnSOD Val16Ala polymorphism and serum TAC and their interactions in relation to CKD in T2DM patients.

## Methods

### Study population

Subjects were participants of Tehran Lipid and Glucose Study (TLGS), an ongoing population-based cohort study conducted to determine the risk factors of non-communicable diseases in a sample of the capital of Iran residents. The first phase of the TLGS was conducted from 1999 to 2001 including 15,005 subjects, aged ≥3 years, and follow-up examinations have been conducted every 3 years (2002–2005; 2006–2008; 2008–2011 and 2011–2014) to identify newly developed diseases. Details of this ongoing cohort study have been published extensively [21, 22].

The current nested case-control study was based on World Health Organization diagnostic criteria [23]*.* A total of 280 T2DM patients in the fifth phase (2011–2014) (*n* = 965) were randomly selected. Inclusion criteria were: having T2DM for at least 5 years; being ≥25 years of age; having no history of cardiovascular, chronic liver or gastrointestinal disease or cancer; no use of glucocorticoids; GFR < 60 ml/min/1.73 m^2^ (defined as CKD stages 3–5) and; no known history of dialysis or kidney transplantation. Each case was individually randomly matched to a control by age (±5 years), sex, duration of T2DM and a GFR of ˃ 60 ml/min/1.73 m^2^ (defined as non-CKD stages 1 and 2). A total of 560 T2DM subjects (280 non-CKD controls and 280 CKD cases) were selected from TLGS phase 5.

The study protocol was approved by the ethical committee of the Research Institute for Endocrine Sciences, Shahid Beheshti University of Medical Science, Tehran, Iran. Written informed consent was obtained from each subject.

### Measurements

Details of data collection in the TLGS have been previously published. In brief, a standard procedure was used for evaluation of anthropometric values. The body mass index (BMI) was calculated. Blood pressure was measured twice, at baseline and after 15 min of rest and the mean of the measurements was reported. Demographic factors were also measured at the study onset using a standard and validated questionnaire [21].

### Laboratory biochemical measurements

A standard colorimetric Jaffe-Kinetic reaction kit (Pars Azmon; Iran; with inter- and intra-assay CVs of 2.5% and 1.9%, respectively, and sensitivity of 0.2 mg/dl) was used to measure serum creatinine (SCr). GFR of each participant was calculated based on SCr, age, and sex using the Modification of Diet in Renal Disease (MDRD) equation: GFR = 186 × (SCr)-1.154 × (Age)-0.203 × (0.742 if female) × (1.210 if black) (24). GFR and SCr are expressed as ml/min per 1.73 m^2^ and mg/dl, respectively, in this equation.

Fasting blood samples were taken after 12–14 h of overnight fasting. The enzymatic colorimetric method with glucose oxidase was used to measure the fasting plasma glucose. Next, 75 g of glucose was administered orally to measure the 2-h plasma glucose (2 h-PG) and plasma glucose.

Triglycerides (TG) and high-density lipoprotein cholesterol (HDL-C) were measured using enzymatic colorimetric method and phosphotungstic acid, respectively. HDL-C measurement was done after precipitation of apolipoprotein B.

Low-density lipoprotein cholesterol (LDL-C) concentrations in samples with serum triglyceride levels of < 400 mg/dl were calculated using Friedewald’s equation. Analysis was performed using standard kits (Pars Azmoon; Iran) and a Selectra 2 auto-analyzer (Vital Scientific; The Netherlands). The inter- and intra-assay coefficient of variation of all assays was < 5%. Simultaneous to sample collection, blood analysis was done in the TLGS research laboratory.

Serum TAC was determined by ferric-reducing antioxidant power assay, using TAC Assay Kit (ZellBio GmbH, Ulm, Germany; Cat.No.: ZB-TAC-A96/48) with 0.1 mM (100 μmol /L) sensitivity. The assay range was 0.125–2 mM (125–2000 μmol /L).

### Genetic analysis

Buffy coats were separated from non-coagulated whole-blood samples and stored at − 80 °C until processing for genotyping of the MnSOD Val16Ala (rs4880) polymorphism. The proteinase K/salting-out method [24] was used to extract DNA from the buffy coat. Tetra-Primer ARMS-PCR assay was administered with minor changes as described by Ruiz-Sanz et al. [25]. Two primer pairs were employed to intensify and designate the genotype of a DNA fragment, including the Ala16Val SNP in the human MnSOD sequence. The 3′-end of the allele-specific primers were underlined F1 (forward) 5´-CACCAGCACTAGCAGCATGT-3′; F2 (forward) 5´-GCAGGCAGCTGGCTaCGGT-3′; R1 (reverse) 5´-ACGCCTCCTGGTACTTCTCC-3′ and; R2 (reverse) 5´-CCTGGAGCCCAGATACCCtAAAG-3′. Underlined lowercase bases denote the incongruence present.

PCR was conducted in a total volume of 40 ml containing 20–40 ng of genomic DNA as the template, 0.5 mM of each primer, 100 mM of each dNTP, 1.25 mM of MgCl2, PCR buffer (20 mM Tris-HCl at pH 8.4 and 50 mM KCl), 5% dimethyl sulfoxide (DMSO) and 1.25 units of DNA polymerase. PCR amplification was conducted with primary denaturation at 94 °C for 7 min, followed by 35 cycles of 60 s of denaturation at 94 °C, 20 s of annealing at 60 °C, 30 s of extension at 72 °C and an additional 7 min extension at 72 °C at the end of the final cycle. Next, 6 ml of loading buffer was mixed with a 20-ml aliquot of the PCR products and resolved by electrophoresis on 1.5% agarose gel. Three distinctive bands in heterozygotes (514, 366 and 189 bp) were generated from this procedure and two bands in homozygotes (Val/Val in bands of 514 and 189 bp and Ala/Ala in bands of 514 and 366 bp). The 514, 366 and 189 bp fragments were directly sequenced to validate the accuracy of genotype scoring using tetra-primer ARMS-PCR. Ten per cent of the samples were sequenced by the Pasteur Institute of Iran, using ABI Genetic Analyzer 3130 and confirmed the accuracy of the MnSOD genotyping methods.

### Statistical analysis

All continuous data with a normal distribution are expressed as mean ± SD. Skewed parameters are expressed as median (25–75%), and categorical variables are expressed as frequency and percentage. Differences in continuous variables were assessed using a t-test or non-parametric Mann–Whitney U test. Differences in categorical variables were assessed using the chi-square test. Allele frequency and genotype frequency were calculated using Power Marker v.3.25 [26]. The Hardy-Weinberg equilibrium, which indicates the absence of discrepancy between genotype and allele frequencies, was checked using the chi-square statistic. A multivariate logistic regression model was used to estimate the odds ratios (ORs) and the corresponding 95% confidence intervals (CIs) of CKD-related factors.

To calculate the interaction of the MnSOD Val16Ala SNP with quartiles of serum TAC in relation to CKD, conditional logistic regression was used. TAC values were categorized into quartiles computed among controls. The results were two likelihood scores with and without the interaction terms. The likelihood ratio test was used to determine the *p*-value for interaction.

To generate ORs for CKD, conditional logistic regression was used in individual carriers or non-carriers of the risk allele (Ala/Ala+Ala/Val and Val/Val) across quartiles of serum TAC (Q1-Q4). As the reference group, the lowest quartile of TAC and the homozygote group (Val/Val) were explored. CKD-associated variables, including BMI, energy intake, total cholesterol, LDL-cholesterol, triglycerides, systolic and diastolic blood pressure, smoking status and medications (anti-diabetic medication (oral and insulin), lipid drugs and anti-hypertensive drugs (diuretic drugs, beta-blockers, ACE-inhibitors, Calcium channel blockers, Angiotensin II receptor antagonists and vasodilator drugs)) included adjusted ORs. The age, sex and duration of T2DM were not adjusted as they were matched at the beginning of the study. Two-tailed *p*-values of < 0.05 were considered statistically significant. Statistical analysis was performed using STATA v.14.0 statistical package or SPSS (ver. 22.0; IBM).

## Results

Table 1 summarizes the general characteristics of the case and control participants. The non-CKD subjects had higher GFR and lower Cr values than the CKD participants. There were no significant differences in all other characteristics between groups. The median serum TAC in the CKD group of patients was 920 μmol/l and was significantly lower (*p* < 0.001) than for the non-CKD group (1045 μmol/l).

**Table 1:** Comparison of characteristics between subjects with and without CKD (*n* = 560)

	CKD (*n* = 280)	Non-CKD (n = 280)	*P* value
GFR (ml/min/1.73m^2^)	54.03 (12.81)	67.54 (6.45)	*<0.001*
Age (y)	64 (12.50)	63 (13.75)	0.154
Sex (Male/Female)	105/175	105/175	–
Duration of diabetes (y)	11.99 (6.4)	11.87 (4.72)	0.417
Body mass index (kg/m^2^)	28.72 (9.9)	28.47 (3.88)	0.554
Systolic blood pressure (mmHg)	13 (3)	13 (3)	0.203
Diastolic blood pressure (mmHg)	80 (18)	79 (20)	0.581
Use of anti-hypertensive drugs	157 (56.5%)	124 (44.9%)	0.057
Fasting plasma glucose (mg/dl)	143 (23)	136 (38)	0.078
2 h-PG (mg/dl)	227.16 ± 89.431	209.09 ± 91.73	0.896
Use of glucose-lowering drugs	201 (72.3%)	207 (74.7%)	0.510
Total cholesterol (mg/dl)	184 (59)	187.5 (41)	0.702
HDL cholesterol (mg/dl)	47 (12)	46 (20)	0.914
LDL cholesterol (mg/dl)	102.8 (49)	106.8 (43)	0.426
Triglyceride (mg/dl)	154.5 (124)	148 (127)	0.347
Use of lipid-lowering drugs	125 (45%)	112 (40.4%)	0.459
Creatinine (mg/dl)	1.2 (0)	1 (0)	*<0.001*
FRAP (μmol/L)	920 (252.5)	1045 (217.5)	*<0.001*
Current or pervious smoking	19 (6.8%)	14 (5%)	0.375
Hyperlipidemia	48 (17.1%)	47 (16.8%)	0.514
Hypertension	32 (11.4%)	48 (17.1%)	0.138
Obesity (BMI ≥ 30)	103 (36.8%)	104 (37.1%)	0.986

Results are expressed as median (IQR), n (%) or mean ± SD. Mann–Whitney U test, t-test or Chi square test P value. CKD = chronic kidney disease. CKD was defined as estimated glomerular filtration rate < 60 ml/min/1.73 m2. *GFR* glomerular filtration rate, *HDL* high density lipoprotein, *LDL* low density lipoprotein, *FRAP* Ferric Reducing Ability of Plasma. The values given in italic in *P*-value column are significant

### Association of MnSOD Val16Ala (rs4880) polymorphism with CKD

The rs4880 polymorphism was in the Hardy–Weinberg equilibrium. In non-CKD subjects, the frequency of the Ala allele was 47.12% and in the CKD cases it was 37.4% (*p* = 0.004). The genotype frequencies for the control and case groups were as follows: Val/Val: 72.5% and 63.2%, respectively; Ala/Ala+Ala/Val: 20.4% and 30.4%, respectively (*p* = 0.001; Table 2). The Ala allele of the Val16Ala polymorphism is associated with a lower risk of CKD (odds ratio (OR) of 0.53; 95% confidence interval (CI) of 0.35–0.82; p = 0.004). Comparing homo and heterozygote genotypes, Ala homozygote genotype (Ala/Ala) indicate a lower risk of CKD compared to the Val homozygote genotype reference group (OR: 0.30; 95% CI: 0.16–0.57; p = 0.001; Table 2). Under the dominant genetic model, Ala allele carriers (Ala/Ala+Ala/Val) indicate a lower risk of CKD compared to the Val homozygote genotype reference group (OR: 0.55; 95% CI: 0.36–0.84; *p* = 0.006; Table 2).

**Table 2:** Adjusted OR for the CKD according to genotype, dominant model and allele of MnSOD Val16Ala genotype

Polymorphism	Genotype	CKD N (%)	Non-CKD N (%)	OR (95% CI)	*P* Value
MnSOD Val16Ala (rs4880)	Genotype				
	Val/Val	85 (30.4%)	57 (20.4%)	1 (ref.)	
	Ala/Val	158 (56.4%)	161 (57.5%)	0.65 (0.44–0.98)	0.041
	Ala/Ala	19 (6.8%)	42 (15%)	0.30 (0.16–0.57)	0.001
	Dominant Genotype Model				
	Val/Val	177 (63.2%)	203 (72.5%)	1 (ref.)	0.006
	Ala/Ala + Ala/Val	85 (30.4%)	57 (20.4%)	0.55 (0.36–0.84)	
	Allele				
	Val	328 (62.6%)	275 (52.88%)	1 (ref.)	0.004
	Ala	196 (37.4%)	245 (47.12%)	0.53 (0.35–0.82)	

ORs (odds ratios and 95% confidence intervals) were calculated using multivariate logistic regression analysis adjusted for BMI, energy intake, total Cholesterol, LDL-Cholesterol, triglyceride, Systolic and Diastolic blood pressure, smoking status and medication; *CKD* chronic kidney disease. CKD was defined as estimated glomerular filtration rate < 60 ml/min/1.73 m2. *eGFR* estimated glomerular filtration rate, *MnSOD* Manganese superoxide dismutase

### Interaction of MnSOD Val16Ala (rs4880) with serum TAC in relation to CKD

A possible interaction between MnSOD Val16Ala polymorphism and serum TAC for the risk of CKD was tested for the T2DM patients. Table 3 presents the adjusted OR for CKD risk based on quartile classification of serum TAC by the dominant model of MnSOD Val16Ala genotypes. Among Ala allele carriers (Ala/Ala+Ala/Val), a decreased risk of CKD was associated with being in the highest quartile of serum TAC over those in the lowest quartile (p-trend = 0.002). No significant association was found between serum TAC and risk of CKD (p-trend = 0.055) in the Val/Val genotype carriers of MnSOD Val16Ala (rs4880; Table 3). No significant interaction was observed between the MnSOD gene Val16Ala polymorphism and serum TAC in relation to CKD in T2DM patients.

**Table 3:** Adjusted OR for the CKD according to quartile (Q) classification of TAC by the genotype and dominant model of MnSOD Val16Ala genotype

MnSOD Val16Ala (rs4880)	OR (95% CI) quartiles				P for trend	P for interaction
	Q1	Q2	Q3	Q4		
Genotype						
Val/Val (*n* = 142)	1 (ref.)	1.79 (0.66–4.83)	2.02 (0.71–5.72)	0.97 (0.36–2.6)	0.055	0.082
Ala/Val (*n* = 319)	2.33 (1.18–4.59)	3.48 (1.83–6.63)	6.38 (3.33–13.2)	4.47 (2.64–7.58)	0.063	
Ala/Ala (*n* = 61)	1.58 (0.33–7.55)	4.75 (0.99–22.67)	4.75 (0.91–24.55)	1.78 (1.06–2.99)	0.133	
Dominant Model						
Val/Val (n = 142)	1 (ref.)	1.79 (0.66–4.83)	2.02 (0.71–5.72)	0.97 (0.36–2.6)	0.055	0.254
Ala/Ala+Ala/Val (*n* = 380)	1.98 (0.82–4.78)	1.13 (0.48–2.65)	0.66 (0.28–1.59)	0.3 (0.12–0.72)	0.002	

ORs (odds ratios and 95% confidence intervals) were calculated using the conditional logistic regression model, adjusted for BMI, energy intake, total Cholesterol, LDL-Cholesterol, triglyceride, Systolic and Diastolic blood pressure, smoking status and medication. The lowest quartile of serum TAC and homozygote genotype with major allele (Val/Val) were used as the reference group

## Discussion

Val16Ala (rs4880) is the most studied MnSOD gene polymorphism for diseases related to including T2DM and its kidney complications [4, 17, 18]. MnSOD Val16Ala polymorphism affects the processing efficiency of the antioxidant enzyme by causing changes in its conformation leading to less efficient mitochondrial transportation of the Val form, which may reduce the MnSOD activity and concentration in the mitochondria and results in inefficient targeting of MnSOD. Lowered resistance to oxidative stress and a decrease and mistargeting of MnSOD activity has been observed more often in homozygous Val/Val than in patients with other MnSOD variants [27, 28]. T2DM patients that are Val carriers can develop hyperglycemia and activation of stress-sensitive signaling pathways by reactive metabolites causing oxidative stress [7]. ROS overproduction and increased oxidative stress have been shown to participate actively in the pathogenesis and progression of T2DM renal complications and CKD [29–31].

The purpose of this study was to clarify the relationship between MnSOD Val16Ala polymorphism and serum TAC in patients with a risk of CKD in T2DM patients. The interaction of the serum TAC level and genetic variants of rs4880 in relation to CKD risk was also examined. The association of MnSOD val16Ala polymorphism has been shown to decrease the risk of CKD and GFR [17, 32–34]. The present study is the first to examine the role of rs4880 in the risk of CKD in T2DM patients and the effect of its interaction with TAC level.

The results show that patients with the Ala/Ala+Ala/Val genotype exhibited lower CKD risk than those with the Val/Val genotype. The Ala allele resulted in higher MnSOD level and increased degradation of ROS, reducing the oxidative stress burden in the carriers [35]. Despite inconsistencies [33, 34], this is in line with results from previous studies which have suggested that the Ala allele carriers have lower risk of decline in kidney function and progression of CKD [17, 32]. Discrepancies among available studies might be explained by differences in disease status and the ethnic backgrounds of the study populations. Studies which are consistent with the present study have reported a relationship between functional impairment of the MnSOD gene with increased risk of renal complications in T2DM patients such as diabetic albuminuria [36, 37] and nephropathy [18, 36, 38]*,* the leading causes of CKD [17, 39].

The serum TAC level decreases significantly in T2DM patients and it has been suggested that the risk of T2DM is lower in subjects with higher serum TAC levels [4, 8, 40, 41]. Low TAC levels associated with hyperglycemia and impaired metabolic status in these patients could exacerbate oxidative stress and increase damage [42, 43]. The current study found a significant decrease in total antioxidant level among T2DM CKD cases at 920 (450–1880 μmol/l) compared to the T2DM non-CKD controls at 1045 (460–1740 μmol/l). Serum total antioxidant activity against oxidative stress to decrease and control damage could result in the decreased TAC level among CKD subjects. Decreases in TAC level due to abnormal lipid peroxidation could also accelerate glomerular damage [41].

Studies have shown that environmental factors potentially can modulate the genetic association between MnSOD Val16Ala and the risk of developing disease or dysfunction which interferes with the redox balance [4, 13]. The effect of the interaction between this MnSOD SNP and antioxidant status on cancer risk has been reported for cervical and breast cancer [13, 44, 45].

An increase in the quartile of the serum TAC participants with the Ala/Ala+Ala/Val genotype was found to decrease CKD risk. However, in T2DM patients, there was no significant interaction between MnSOD Val16Ala SNP and the TAC level in relation to CKD. More studies are needed to investigate the role of factors directly related to oxidative stress, such as serum TAC in T2DM complications and CKD- formation and progression.

The strengths of the current study were the sampling of the prospective TLGS study participants for long-term follow-up, individually-matching controls by age, sex and diabetes duration, the drug history of participants and extensive adjustment for potential CKD confounders. Although limited potential for selection bias is of a concern in nested case control studies, applying individual matching by our study inclusion criteria in our sampling, we tried to minimize it. One other limitation is the lack of generalization due to selection of the population from TLGS participants.

## Conclusion

The findings demonstrate that the MnSOD Val16Ala polymorphism and serum TAC level are associated with CKD independent of other known risk factors in type 2 diabetic patients. The Ala allele appears to provide resistance to CKD in T2DM patients. Decreased median serum TAC was observed in the CKD patients. No significant interaction was found between MnSOD Val16Ala polymorphism and serum TAC in relation to CKD.

## Abbreviation

2 h-PG: 2-h plasma glucose
BMI: body mass index
CI: confidence interval
CKD: Chronic kidney disease
DMSO: Dimethyl sulfoxide
FFA: free fatty acid
GFR: glomerular filtration rate
HDL-C: high-density lipoprotein cholesterol
LDL-C: Low-density lipoprotein cholesterol
MDRD: Modification of Diet in Renal Disease
MnSOD: Manganese superoxide dismutase
OR: odds ratio
ROS: reactive oxygen species
SCr: serum creatinine
SNPs: single-nucleotide polymorphisms
T2DM: Type 2 diabetes mellitus
TAC: Total antioxidant capacity
TG: Triglycerides
TLGS: Tehran Lipid and Glucose Study
